# Follow-Up of Patients With Axial Spondyloarthritis in Specialist Health Care With Remote Monitoring and Self-Monitoring Compared With Regular Face-to-Face Follow-Up Visits (the ReMonit Study): Protocol for a Randomized, Controlled Open-Label Noninferiority Trial

**DOI:** 10.2196/52872

**Published:** 2023-12-27

**Authors:** Inger Jorid Berg, Anne Therese Tveter, Gunnstein Bakland, Sarah Hakim, Eirik K Kristianslund, Siri Lillegraven, Gary J Macfarlane, Ellen Moholt, Sella A Provan, Joseph Sexton, Emil EK Thomassen, Annette De Thurah, Laure Gossec, Espen A Haavardsholm, Nina Østerås

**Affiliations:** 1 Center for Treatment of Rheumatic and Musculoskeletal Diseases (REMEDY) Diakonhjemmet Hospital Oslo Norway; 2 Faculty of Health Sciences, Department of Rehabilitation Science and Health Technology Oslo Metropolitan University Oslo Norway; 3 Department of Rheumatology University Hospital of North Norway Tromsø Norway; 4 Institute of Clinical Medicine, Faculty of Health Sciences UiT The Arctic University of Tromsø Tromsø Norway; 5 Aberdeen Centre for Arthritis and Musculoskeletal Health (Epidemiology Group) University of Aberdeen Aberdeen United Kingdom; 6 Section for Public Health Inland Norway University of Applied Sciences Elverum Norway; 7 Department of Rheumatology Aarhus University Hospital Aarhus Denmark; 8 Department of Clinical Medicine Aarhus University Aarhus Denmark; 9 INSERM, Institut Pierre Louis d'Epidémiologie et de Santé Publique Sorbonne Université Paris France; 10 Rheumatology Department Assistance Publique des Hopitaux de Paris Pitié-Salpêtrière Hospital Paris France

**Keywords:** spondyloarthritis, inflammatory joint disease, telemedicine, remote monitoring, remote care, self-management, randomized controlled trial, cost-effectiveness

## Abstract

**Background:**

Patients with chronic inflammatory joint diseases such as axial spondyloarthritis have traditionally received regular follow-up in specialist health care to maintain low disease activity. The follow-up has been organized as prescheduled face-to-face visits, which are time-consuming for both patients and health care professionals. Technology has enabled the remote monitoring of disease activity, allowing patients to self-monitor their disease and contact health care professionals when needed. Remote monitoring or self-monitoring may provide a more personalized follow-up, but there is limited research on how these follow-up strategies perform in maintaining low disease activity, patient satisfaction, safety, and cost-effectiveness.

**Objective:**

The Remote Monitoring in Axial Spondyloarthritis (ReMonit) study aimed to assess the effectiveness of digital remote monitoring and self-monitoring in maintaining low disease activity in patients with axial spondyloarthritis.

**Methods:**

The ReMonit study is a 3-armed, single-site, randomized, controlled, open-label noninferiority trial including patients with axial spondyloarthritis with low disease activity (Ankylosing Spondylitis Disease Activity Score <2.1) and on stable treatment with a tumor necrosis factor inhibitor. Participants were randomized 1:1:1 to arm A (usual care, face-to-face visits every sixth month), arm B (remote monitoring, monthly digital registration of patient-reported outcomes), or arm C (patient-initiated care, self-monitoring, no planned visits during the study period). The primary end point was disease activity measured with the Ankylosing Spondylitis Disease Activity Score, evaluated at 6, 12, and 18 months. We aimed to include 240 patients, 80 in each arm. Secondary end points included other measures of disease activity, patient satisfaction, safety, and cost-effectiveness.

**Results:**

The project is funded by the South-Eastern Norway Regional Health Authority and Centre for the treatment of Rheumatic and Musculoskeletal Diseases (REMEDY), Diakonhjemmet Hospital, Norway. Enrollment started in September 2021 and was completed with 242 patients by June 2022. The data collection will be completed in December 2023.

**Conclusions:**

To our knowledge, this trial will be among the first to evaluate the effectiveness, safety, and cost-effectiveness of remote digital monitoring and self-monitoring of patients with axial spondyloarthritis compared with usual care. Hence, the ReMonit study will contribute important knowledge to personalized follow-up strategies for patients with axial spondyloarthritis. These results may also be relevant for other patient groups with inflammatory joint diseases.

**Trial Registration:**

ClinicalTrials.gov NCT05031767; hpps://www.clinicaltrials.gov/study/NCT05031767

**International Registered Report Identifier (IRRID):**

DERR1-10.2196/52872

## Introduction

### Background

Chronic inflammatory joint diseases (IJDs), including axial spondyloarthritis (axSpA), rheumatoid arthritis (RA), and psoriatic arthritis (PsA), often affect young patients. Most patients are treated with disease-modifying antirheumatic drugs (DMARDs). These patients are scheduled for regular follow-up visits in specialist health care, which can be time-consuming, especially for those active in the workforce. Patients have also reported that in this mode of regular follow-up the health care professionals (HPs) only see a narrow timeframe of their disease course, with important changes often taking place between visits [1].

### Details on axSpA

The primary effects of axSpA occur in the sacroiliac joints and the spine, where the main concerns are back pain and spinal stiffness. Some patients experience arthritis and enthesitis and may also have extramusculoskeletal manifestations, such as uveitis, psoriasis, and inflammatory bowel disease [2]. The prevalence of axSpA is 0.3% to 1.4%, and the onset is typically in the third decade of life, with an equal gender distribution [3]. As a consequence of spinal inflammation, structural damage may occur, resulting in limited spinal mobility [3]. Furthermore, high disease activity may affect health-related quality of life with work impairment and a substantial socioeconomic load [4].

The treatment of axSpA is individualized according to the patient’s needs, disease manifestations, and level of inflammation. The treatment goals include symptomatic relief, maintaining flexibility of the spine, maintaining or improving function and work ability, and preventing disease-related complications [2,5]. First-line treatment consists of exercises and the use of nonsteroidal anti-inflammatory drugs (NSAIDs). Regular exercises and physical activity are recommended for symptom relief and reduced disease activity [5,6]. A recent study demonstrated that patient-reported flares are associated with impaired physical activity [7]. NSAIDs also reduce pain and stiffness, with doses adjusted to symptom severity [2,5]. Second-line treatment includes the use of DMARDs, usually tumor necrosis factor inhibitors (TNFis) [5], which in most patients leads to a rapid, substantial, and sustained improvement of symptoms, inflammation, and physical function [2], enabling participation in the workforce and in daily activities [4,5].

Follow-up of patients with axSpA treated with TNFi includes long-term monitoring of disease activity, clinical findings, and laboratory tests, with a treat-to-target strategy usually aimed at inactive disease or low disease activity as measured by the Ankylosing Spondylitis Disease Activity Score (ASDAS) <2.1 [5,8,9]. Patients are reviewed regularly during face-to-face visits by a rheumatologist or a rheumatology nurse in specialist health care. The frequency of monitoring is individualized depending on the disease severity. At most clinics, the patients can request extra visits when they experience increased disease activity (flares).

In recent years, technology has opened up for remote monitoring through the digital collection of patient-reported outcomes (PROs) and the use of tools for supporting medical decisions. Patients may receive remote care or self-monitor their disease and receive remote support from HPs when needed [10]. Together with treat-to-target strategies, remote care may improve clinical outcomes [11]. The timing of face-to-face or digital visits may be individualized [11], which may increase patient satisfaction. Patients living in both rural and urban areas with limited access to health care services may experience increased access to health care services if they are offered digital follow-up [12,13]. Remote monitoring and self-monitoring may be a step toward more personalized and potentially better follow-up of patients with axSpA. Remote care may reduce the need for visits to specialist health care and thus be cost-effective.

### Prior Work

The effect, user satisfaction, and resource use of new follow-up regimens should be evaluated before their implementation in usual clinical care. Previous studies on remote monitoring in patients with other chronic diseases, such as cardiovascular disease, lung disease, and diabetes mellitus, have shown beneficial results on clinical outcomes [14]. However, a recent systematic review found little or no effect of resource use in specialist health care services [15].

There is limited research on the remote follow-up of IJDs [16-18]. A few studies on patients with RA and PsA in low disease activity or remission found similar disease activity in patients followed by remote monitoring and usual care [10,19], and patient satisfaction with remote care was high [1,20]. Furthermore, patients have reported that self-monitoring of the disease enables them to see the connection between behavioral factors (eg, taking medications and lifestyle choices) and disease concerns [1]. However, a survey regarding the use of digital technology during the COVID-19 pandemic revealed that HPs considered remote care inferior to face-to-face visits [21]. In a recent study, both patients and HPs considered remote care inferior to face-to-face visits, but remote care was convenient for patients in relation to reduced traveling and waiting times [22]. In studies on patients with RA and PsA, the relative cost-effectiveness has shown conflicting results [23,24]. There is limited research on the sustainability of remote follow-up and self-monitoring in patients with axSpA.

### Aim of This Study

**Figure 1:**
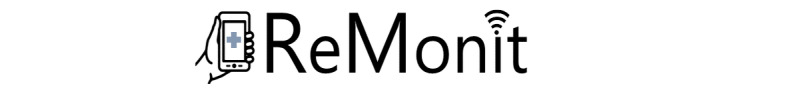
Logo of the Remote Monitoring in Axial Spondyloarthritis (ReMonit) study.

The primary aim of the Remote Monitoring in Axial Spondyloarthritis (ReMonit; Multimedia Appendix 1) study is to determine whether 2 new follow-up strategies for patients with axSpA—remote monitoring or patient self-monitoring—are noninferior to the conventional follow-up strategy with regular prescheduled face-to-face visits to maintain low disease activity over time. Second, we aim to evaluate patient satisfaction, HP satisfaction, safety, and cost-effectiveness of the 3 follow-up strategies (Figure 1). Our hypothesis is that remote monitoring and patient self-monitoring are noninferior to traditional follow-up with regular face-to-face visits in maintaining low disease activity and that these new follow-ups are associated with patient and HP satisfaction, safety, and cost-effectiveness.

Comprehensive data collection allows for analyses of willingness to use, adherence to remote care among patients, fluctuations in disease activity, and associations between disease activity and physical activity.

## Methods

### Study Design

The ReMonit study is a 3-armed, single-center, parallel-group, noninferiority randomized controlled trial (RCT) designed to investigate whether 2 new follow-up strategies for patients with axSpA are noninferior to conventional follow-up for maintaining stable low disease activity (NCT05031767; registered on September 2, 2021).

### Study Settings, Population, and Recruitment

Patients were recruited between September 2021 and June 2022 from the outpatient clinic at the Division of Rheumatology and Research, Diakonhjemmet Hospital, Oslo, Norway. Eligible patients (axSpA with stable treatment with TNFi and low disease activity [ASDAS <2.1]) were identified from the waiting list for regular follow-up visits and were sent information about the ReMonit study and a consent form, along with an invitation to a visit with a rheumatologist. At the visit, patients willing to participate were screened according to the inclusion and exclusion criteria (Textbox 1).

**Inclusion and exclusion criteria.**

**Inclusion criteria**

- Participants were eligible to be included in the study only if all the following criteria applied
  Male or female aged >18 years of age at screening
  Diagnosis of axial spondyloarthritis (axSpA) according to the Assessment of Spondyloarthritis International Society classification criteria for axSpA [25]
  Stable medical treatment with tumor necrosis factor inhibitors for the last 6 months
  Inactive disease or low disease activity (Ankylosing Spondylitis Disease Activity Score <2.1) at inclusion
  Capable of understanding the Norwegian language and of providing informed consent.

**Exclusion criteria**

- Medical conditions
  Major comorbidities, such as severe malignancies, severe diabetes mellitus, severe infections, uncontrollable hypertension, severe cardiovascular disease (New York Heart Association class III or IV), severe respiratory diseases, or cirrhosis
  Indications of active tuberculosis
  Pregnant or nursing
- Diagnostic assessments
  Abnormal renal function, defined as serum creatinine >142 µmol/L in female participants and >168 µmol/L in male participants or glomerular filtration rate <40 mL/min/1.73 m2
  Abnormal liver function (defined as alanine transaminase >3 times the upper normal limit) and active or recent hepatitis
  Leukopenia, thrombocytopenia, or both
- Other
  Severe psychiatric or mental disorders, alcohol abuse or other substance abuse, language barriers or other factors which makes adherence to the study protocol impossible.

### Ethical Considerations

This study was approved by the Norwegian Regional Committee for Medical and Health Research Ethics ( 229187) as well as the data protection official (DS-00372) and the local research committee at Diakonhjemmet Hospital. The ReMonit study was conducted according to the Declaration of Helsinki. The Norwegian Regional Committee for Medical and Health Research Ethics approved the collection of data (age, gender, diagnosis, comorbidities, medication, disease activity, laboratory measurement, level of education, work status, and smoking habits) from nonparticipating patients attending a screening visit, both screening failures, and patients unwilling to participate in the RCT, if the patients gave their informed consent. These data will be used to evaluate potential selection bias in patient recruitment.

### Randomization and Blinding

The included patients were allocated in a 1:1:1 ratio among the 3 study arms (Textbox 2). A statistician and a secretary, not involved in patient screening or enrollment, provided a computer-generated block randomization list (random block size of 6, 9, or 12 patients) and prepared sealed, opaque envelopes containing information on study arm assignment. Participants and treating clinicians are not blinded to the group allocation, but the statistician that will perform the primary outcome analyses is blinded.

**Study arms.**

Arm A (usual care): conventional follow-up strategy with prescheduled face-to-face visits at the hospital every 6 months, with an assessment of blood tests, patient-reported outcomes (PROs), and joint examination

Arm B (remote monitoring): hospital health care professionals (HPs) perform remote monitoring of PROs, the HP contacts patients if the PRO score indicates disease flare or if the patient requests an appointment with HPs

Arm C (patient-initiated care): self-monitoring by the patients with no prescheduled visits or remote monitoring, the HP contacts the patient if the patient requests an appointment with HPs

### Intervention and Follow-Up

**Figure 2:**
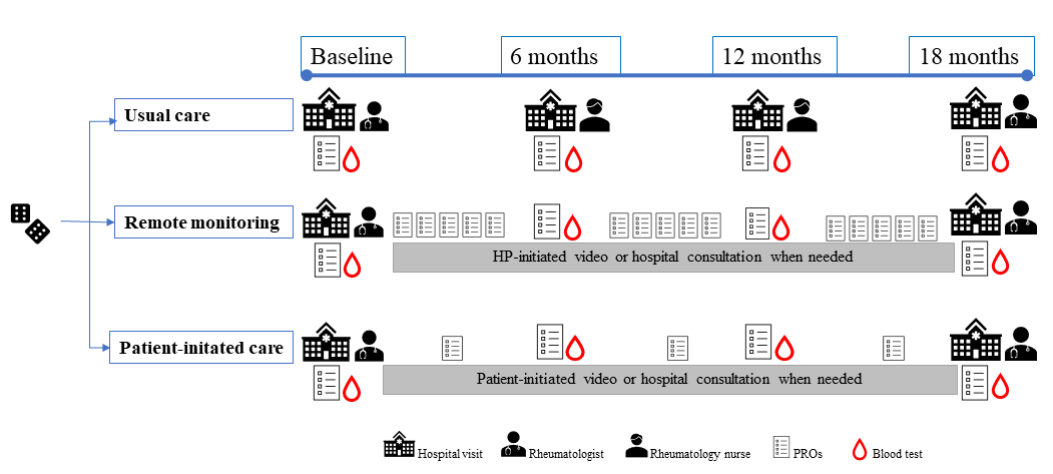
Illustration of the Remote Monitoring in Axial Spondyloarthritis (ReMonit) study design. CRP: c-reactive protein; HP: health care professional; PRO: patient-reported outcome.

The follow-up for arms A, B, and C lasts for 18 months with data collection at baseline and 6, 12, and 18 months (Figure 2).

### Digital Monitoring: MyDignio App and Dignio Prevent Platform

Dignio AS offers digital solutions for remote care in the health care system, is certified according to international standards of information security, and has approval for communication of health data in Norwegian health care services [26]. All communication through the software is encrypted and in compliance with the current legislation.

The patients download the MyDignio app on their smartphone or tablet and can report on PROs and manually report data, such as laboratory measurements, in the app. Several instruments can be integrated into the Dignio system, including measurements of physical activity. The HPs can monitor the patient-reported data and measurements on the web-based Dignio Prevent platform, and PROs and measurements can be displayed as graphs or histograms. The MyDignio app and the Dignio Prevent platform also provides the possibility of asynchronous chats between patients and HPs and synchronous communication with video consultations.

In this study, the patients use MyDignio for reporting PROs in the intervention arms B and C and HPs use Dignio Prevent for monitoring PROs in arm B, as described in detail in subsequent sections. Physical activity registered as steps and heart rate is recorded by a smartwatch connected via Bluetooth to a smartphone or tablet that synchronizes data via MyDignio. We also used Dignio for short communication for the patients in arms B and C, such as to send messages reminding patients on completing regularly reported PROs (arms B and C), regular blood sampling, and a link to a questionnaire every 6 months.

### Description of the Study Arms

#### Usual Care: Arm A

Patients underwent conventional prescheduled face-to-face visits with an experienced rheumatology nurse at 6 and 12 months and with a rheumatologist at 18 months (study end), with a review of disease-related concerns, blood test results, joint examination as well as recording medication use, and adverse events. Patients who are unable to attend to a visit are offered a rescheduled visit. Patients can call the study nurse if they experience significant symptom worsening or adverse events.

#### Remote Monitoring: Arm B

No visits are prescheduled between the baseline visit and study-end visit. Patients download the MyDignio app on their smartphone or tablet and receive a brief introduction from the study coordinator. For monitoring, the patients receive a monthly SMS text message reminder for reporting PROs, which includes the Patient Global Assessment (PGA) and whether they experience a flare (significant worsening of disease-related symptoms). If the PGA is scored ≥3 or they answer “yes” or “uncertain” on the flare question, they are asked to report disease activity using the Bath Ankylosing Spondylitis Disease Activity Index (BASDAI) [27]. These PRO reports are monitored daily (Monday to Friday) by a study nurse via Dignio Prevent, where patients that need attention are highlighted based on predefined values for the outcomes (Table 1). If patients do not report the PROs, Dignio Prevent alerts the study coordinator who sends a reminder using Dignio. Patients can send messages to the HPs through the app or call the study nurse if they experience significant symptom worsening or adverse events.

**Table 1:** Indication for the health care professionals (HPs) to contact a patient in the remote monitoring study arm.

Indication	Definition	Action
Significant worsening	Yellow flag: BASDAI^a^ ≥4 in the monthly PRO^b^ reporting Red flag: BASDAI ≥8 in the monthly PRO reporting	The study nurse contacts the patient to evaluate if a visit is needed
The patient has requested to be contacted by the HP	The patient sends a message in MyDignio app asking to be contacted by HP	The study nurse contacts the patient to evaluate if a visit is needed

^a^BASDAI: Bath Ankylosing Spondylitis Disease Activity Index.

^b^PRO: patient-reported outcomes.

#### Patient-Initiated Care: Arm C

No visits are scheduled between the baseline visit and study-end visit. Patients in arm C download the MyDignio app on their smartphone or tablet and receive a brief introduction from the study coordinator. The patients receive an SMS text message reminder for reporting PROs every third month (PGA and patient-reported flare, the same as that reported monthly in arm B), but the PROs are not monitored by HPs. If patients do not report the PROs, Dignio Prevent alerts the study coordinator who sends a reminder using Dignio. Patients can send messages to HPs through the app or call the study nurse if they experience significant symptom worsening or adverse events.

### Medical Treatment

At inclusion, all eligible patients were on stable medical treatment with TNFi for the last 6 months, and TNFi were continued for patients in all 3 study arms. If, during the study, patients experience a minor worsening of disease-related symptoms, they can initiate or intensify treatment with NSAIDs. Patients experiencing a significant worsening of the disease (identified either at a visit with a nurse in arm A, registered by digital monitoring in arm B, or by the patient contacting the study nurse in either of the arms) are offered a face-to-face visit with a rheumatologist within 2 weeks to evaluate the treatment regimen. The dose of TNFi may be adjusted or if TNFi therapy fails, switching to another TNFi (or an interleukin-17 inhibitor) is considered. In the case of switching treatment, the patient is offered a face-to-face follow-up visit 3 months later to ensure the effectiveness and tolerability of the new medication.

### Study Assessments Schedule

Research data are collected throughout the study, separately from the remote monitoring data collected with the My Dignio app in study arm B. The main data collection time points are at baseline and at 6, 12, and 18 months (study end) for all patients (Table 2). Data are collected through interviews by a rheumatologist and a study nurse, self-reports by patients in digital questionnaires, physical examinations by a rheumatologist or a study nurse, laboratory assessments or blood samples, and a physical activity tracker or smartwatch. A qualitative substudy with individual interviews will be conducted after the end of the study.

**Table 2:** Schedule of activities.

Procedure	Screening	Baseline	Intervention period (remote monitoring or visits)					Extra visits and early discontinuation
			Every month	6th-month follow-up	12th-month follow-up	18th month, study end		
Inclusion and exclusion criteria	✓							
Fulfil ASAS^a^ criteria	✓							
Physical examination including the heart and lungs	✓							
Informed consent	✓							
Randomization		✓						
Safety laboratory tests^b^		✓		✓	✓	✓	✓	
CRP^c^ and ESR^d^		✓	✓^e^	✓	✓	✓	✓	
Clinical examination of disease activity, including enthesitis (heel) and peripheral arthritis		✓		✓^f^	✓^f^	✓	✓	
Vital signs		✓						
Medical history		✓						
Demography		✓						
Lifestyle		✓						
eHealth literacy		✓						
Patient Global Assessment		✓	✓^g^	✓	✓	✓	✓	
Patient-reported outcomes		✓	✓^g^	✓	✓	✓	✓	
Medication		✓		✓	✓	✓	✓	
Adverse event review				✓	✓	✓	✓	
Physical activity monitoring			✓^h^	✓^h^	✓^h^			
Reason for discontinuation^i^							✓	

^a^ASAS: Assessment of Spondyloarthritis International Society.

^b^Measured at baseline and every 3 months throughout the study as recommended due to treatment with tumor necrosis factor inhibitors. Blood samples are analyzed by the patient’s general practitioner or at the Diakonhjemmet Hospital, whatever is the most convenient for the patients. The following blood samples will be analyzed: hemoglobin, red blood cells, white blood cells with differential count, platelets, creatinine, and alanine transaminase.

^c^CRP: c-reactive protein.

^d^ESR: erythrocyte sedimentation rate.

^e^Only the subgroup (n=12) in the remote monitoring arm (arm B) that received a home-based C-reactive protein instrument.

^f^At 6 and 12 months, only in the usual care arm (arm A).

^g^Patients in the remote monitoring arm (arm B) will complete a brief questionnaire each month, and patients in the “patient-initiated care” arm (arm C) will complete a brief questionnaire only every 3 months.

^h^Only patients in the remote monitoring arm (arm B) and patient-initiated care arm (arm C) from inclusion or randomization until 12 months.

^i^Only at the early discontinuation visit.

#### Interview With a Rheumatologist and a Study Nurse

Information on medical history and fulfillment of the Assessment of Spondyloarthritis International Society classification criteria is obtained at baseline by a rheumatologist. Information on medication is recorded at baseline, extra visits, study end, and early discontinuation in all groups as well as at the 6- and 12-month visits in arm A. The dosage and frequency of TNFi and concomitant medication is recorded. The use of NSAIDs, oral or injected glucocorticoids, and analgesics for residual pain (paracetamol or opioids) has received particular attention. Adverse events are reported at all follow-up visits. At the end of the study, the patients will be asked about their preference for future follow-up strategies.

#### Digital Questionnaires

All patients completed digital questionnaires at baseline and 6, 12, and 18 months and at extra visits and early discontinuation. Data collections were handled by questionnaires created with Nettskjema, a survey solution developed and hosted by the University of Oslo. At baseline, patients reported demographic data, lifestyle factors (smoking and physical activity), educational level, work status, travel distance, travel time, and way of transport to the hospital. The patients also responded to questions regarding experience with digital technology and eHealth literacy through 20 items from 4 domains of the eHealth Literacy Questionnaire; domain 1, using technology to process health information; domain 3, the ability to actively engage in digital services; domain 4, feel safe and in control; and domain 5, motivated to engage with digital services (response options ranged from 1—strongly disagree, to 4—strongly agree) [28].

Several PROs on disease activity, functional status, sleep quality, health-related quality of life, and patient satisfaction with care and physical activity are reported at baseline and 6, 12, and 18 months; extra visits; and early discontinuation (Table 3). The patients also report, in line with the Assessment of Spondyloarthritis International Society recommendations [29], on the use of medication for spondyloarthritis (TNFi, NSAIDs, and steroids) and if TNFi has been taken as recommended.

**Table 3:** Patient-reported secondary outcomes at baseline and 6-, 12- and 18-month data collection time points, at extra visits, and at early discontinuation.

Patient-reported outcome	Measurement scale
PGA^a^	NRS^b^ 0-10
Pain assessment	NRS 0-10
Joint pain assessment	NRS 0-10
BASDAI^c^ [27]	6 items, NRS 0-10
BASFI^d^ [30]	10 items, NRS 0-10
Flare^e^	Yes, no, or uncertain If yes or uncertain, which date the flare occurred and number of days it lasted
Change in disease activity last 6 months^e^	7-point scale ranging from “much worse” to “much better”
Change in activity impairment last 6 months^e^	7-point scale ranging from “more activity impairment” to “improved ability to performed daily activities”
WPAI^f^ item no. 6: activity impairment last week [31]	1 item, NRS 0-10
Pittsburgh Sleep Quality Index: sleep disturbances due to pain [32]	1 item 4-point scale from “not during the past month” to “3 or more times a week”
EQ-5D-5L [33,34]	5 items 5-point scale + Health index, VAS^g^ 0-100
The patients’ satisfaction with care [35]	1 item 5-point response options ranging from “very satisfied” to “very dissatisfied”
Patient-reported physical activity [36]	3 items Frequency (never, less than once, once, 2 to 3 times, or ≥4 times per week) Duration (less than 15, 15-30, 31-60, or ≥60 min) Intensity (no sweat, sweat, or exhausted)

^a^PGA: Patient Global Assessment.

^b^NRS: numeric rating scale.

^c^BASDAI: Bath Ankylosing Spondylitis Disease Activity Index.

^d^BASFI: Bath Ankylosing Spondylitis Functional Index.

^e^Not reported at baseline.

^f^WPAI: Work Productivity and Activity Impairment questionnaire.

^g^VAS: visual analog scale.

In arm A, patients receive a link to the digital questionnaires by SMS text messages a few days ahead of the 6-, 12-, and 18-month visits. Participants in arms B and C receive a digital reminder through the MyDignio app at the time of data collection at 6 and 12 months, with a link to the digital questionnaire, but the link is sent by SMS text messages for the 18-month visit. The study personnel carefully monitor the data collection to ensure completeness and contact nonresponders by SMS text messages or phone calls.

#### Physical Examinations

Vital signs are examined at baseline (Table 2). A joint examination is performed at baseline, extra visits, study end, and early discontinuation in all arms and at 6- and 12-month visits in arm A.

#### Laboratory Assessments

Standard care blood samples (Table 2; hemoglobin, red blood cells, white blood cells with differential count, platelets, creatinine, and alanine transaminase) are analyzed as a safety procedure when using TNFi. Patients in arms B and C are asked to report the erythrocyte sedimentation rate (ESR) and C-reactive protein (CRP) values at 6 and 12 months if measurements are performed by their general practitioner. A subgroup of 12 patients in arm B are asked to perform home-based measurements of CRP each month, in connection with the monthly reporting of PROs. The CRP level is measured using the QuikRead go instrument [37], and the patients register the CRP value in the MyDignio app.

#### Registration of Physical Activity

The patients in arms B and C are asked to wear a water-resistant smartwatch (Garmin Vivosmart 4) for physical activity monitoring, for example, the number of steps and heart rate during daytime, and data are transferred to Dignio. They are instructed to wear this smartwatch for at least 10 hours during the day, but they can wear it all day and night should they want to. Patients who do not want to wear this smartwatch can still participate in the study in the allocated arm.

#### Qualitative Substudy

A qualitative substudy with observations and individual semistructured interviews will be conducted after the study ends to gain in-depth insight from patients and HPs on their experiences with remote monitoring. We are planning to conduct interviews with 15 to 20 patients and 5 to 8 HPs. The interview will be recorded through Nettskjema and sent encrypted to Services for Sensitive Data at the University of Oslo.

### Outcomes

#### Primary Outcome

The primary outcome of the study is low disease activity, defined as ASDAS <2.1, and will be evaluated by point prevalence across the 6-, 12-, and 18-month data collection time points. ASDAS is calculated from 4 PROs on patients’ experience of back pain, joint pain or swelling, duration of morning stiffness, and PGA during the last week (reported on a visual analog scale, 0-100, 0 being best and 100 being worst) and CRP or ESR, where ASDAS_CRP_ is the preferred measure.

The formula for ASDAS_CRP_ is as follows: 0.12 × back pain + 0.06 × duration of morning stiffness + 0.11 × patient global + 0.07 × peripheral pain or swelling + 0.58×Ln(CRP + 1)). The formula for ASDAS_ESR_ = 0.08 × back pain + 0.07 × duration of morning stiffness + 0.11 × patient global + 0.09 × peripheral pain or swelling + 0.29 × √(ESR) [38,39]. In this study, we will calculate ASDAS_CRP_; however, if CRP is missing and we have the value for ESR, ASDAS_ESR_ will be used.

#### Secondary Outcomes

Secondary outcomes include other measurements of disease activity (eg, ESR, CRP, BASDAI, PGA, change in PGA, pain and joint pain assessment, flare, swollen and tender joint count, presence of heel enthesitis, extramusculoskeletal manifestations, and use of medication), functional status (Bath Ankylosing Spondylitis Functional Index, change in activity impairment, and work productivity and activity impairment item no 6), and sleep quality (Pittsburgh Sleep Quality Index: sleep disturbances due to pain). Furthermore, patient satisfaction with care will be evaluated.

Rates and types of adverse events and serious adverse events are recorded throughout the study at all follow-up visits, extra visits, early discontinuation, and study-end visits by structured interviews and reported in the electronic case report form (eCRF). If an adverse event is considered to be related to the treatment of the patient, the patient’s medical treatment is evaluated by a rheumatologist and changed if needed or recommended. Recording adverse events, use of medications such as painkillers, and possible use of antibiotics can provide information on the safety of the 3 follow-up arms.

#### Substudies

Assessment of the EQ-5D-5L, time for patient being absent from work and potential travel costs related to consultations, time and costs related to patient monitoring (phone calls from patients to nurse or rheumatologist, telephone consultations, video consultations, and face-to-face visits), and health care use (in primary and secondary health care) will provide information on health care use and will allow for evaluation of cost-effectiveness.

All patients will at baseline self-report their experience with digital technology and eHealth literacy. Patients will report on satisfaction with care (Table 3), and patients in arm B will also report on their experiences on reporting monthly disease activity at the 18-month follow-up [40,41].

This qualitative substudy with observations and individual semistructured interviews will provide in-depth insight from patients and HPs on their experiences with remote monitoring.

The close measurement of disease activity by PGA and the monthly CRP for the subgroup in arm B will allow for analyses of fluctuations in disease activity over time. Self-reported physical activity (Table 3) and the measurement of physical activity with smartwatches in arms B and C will provide data to analyze the potential associations between disease activity and physical activity.

#### Linkage to Registers

The ReMonit project has approval to link data with several national registers in Norway: KUHR (register of national financing support for patients in health services in Norway by Norwegian Directorate of Health), FD-trygd (register of work status, sick leave, and disability pension by Statistics Norway), NPR (register on health information on patients having received treatment or waiting for treatment in specialist health care in Norway by Norwegian Directorate of Health), and NorPD (register of prescription of drugs dispensed from pharmacies, Norwegian Prescription Database by Norwegian Institute of Public Health). These data can provide information for the cost-effectiveness analyses.

### Statistics

#### Sample Size and Power Considerations

On the basis of data from the NOR-DMARD database (an ongoing longitudinal observational study of the effectiveness of treatment of IJDs with biological disease-modifying antirheumatic drugs in clinical practice, ClinicalTrials.gov NCT01581294) [42], we expect approximately 88% of the usual care patients to have low disease activity at the end of the study. We estimate that 74 patients in each arm will give a power of 80% to conclude that at least one of the alternative follow-up strategies is noninferior to usual care. This assumes a noninferiority margin of 15%, no actual difference between the groups, and an analysis based only on one time point. By collecting the primary end point at 3 time points (and not only at the end of study) for each participant, the power will increase, and a group size of 80 participants should have sufficient power, even with 15% dropout or missing data.

#### Statistical Analyses

This study will compare the point prevalence of low disease activity (ASDAS <2.1) in the remote monitoring (arm B) and patient-initiated care (arm C) arms to that of usual care (arm A). A monitoring regime will be deemed noninferior to usual care if it can be shown that its low disease activity prevalence is no more than 15 percentage points below that of usual care. In addition, we will test the noninferiority of patient-initiated care to remote monitoring using the same 15% margin. This test will be done only if patient-initiated care is shown to be noninferior to conventional care (a hierarchical test) and thus will not inflate the 5% false positive rate.

The statistical analysis plan will be finalized, signed, and dated before data locking. Demographics, baseline characteristics, efficacy, and safety variables will be summarized using descriptive statistics. Efficacy (both primary and secondary endpoints) and safety analyses will include data from all randomized patients who started the allocated intervention by completing at least one questionnaire and blood test results after randomization, the full analyses set. Robustness analyses will be performed in the per-protocol set, which includes all randomized patients meeting the study entry criteria who followed the study protocol (meeting appointments and reporting data as described in the follow-up regimen of arms A, B, and C).

#### Primary Analyses

The primary end point, the point prevalence of ASDAS <2.1, will be analyzed by mixed effect logistic regression. The analysis will be based on follow-up data from 6-, 12-, and 18- month data collection time points and will include the randomization group as the main covariate. Sensitivity analyses will include additional covariates such as age, gender, and disease characteristics. The estimates of between-group differences in point prevalence will be based on adjusted risk differences [43]. The noninferiority of remote monitoring to usual care will be evaluated by the CI for the between-group difference in low disease activity (point) prevalence, with a similar approach for patient-initiated care versus usual care [44].

#### Secondary Analyses

Analyses of secondary effectiveness end points will be presented using point estimates of between-group differences along with their 95% CIs. Continuous and binary outcomes will be analyzed using mixed effects linear and logistic regression, respectively. Cost-effectiveness of the 2 new follow-up strategies will be evaluated primarily with a health care perspective. If the new follow-up strategies are considered noninferior to usual care, cost-minimization analyses may be relevant. Otherwise, calculations of quality-adjusted life-years, determination of the incremental or decremental cost-effectiveness ratio, and the assessment of cost-effectiveness using the cost-effectiveness acceptability curve may be considered. Details of the analyses will be described in the statistical analyses plan.

#### Missing Data

In the ReMonit study, the primary end point will be collected at 3 time points for each patient, and only patients with at least 1 assessment of the primary end point will be included in the final analyses (the full analyses set). For the main analysis, missing values of the primary end point will not be imputed, as the mixed effect analysis provides unbiased effect estimates, provided the missing data are missing-at-random. Alternative treatments for missing values of the primary end point will be entertained as sensitivity analyses.

### Data Registration, Monitoring, and Storage

Data are reported in the eCRF and stored safely at Services for Sensitive Data provided by the University of Oslo [45]. Guidance on the completion of eCRFs is provided in the investigator brochure and eCRF. The regular self-reported disease activity in arms B and C, the CRP measurements performed by the subgroup of patients, and the data on physical activity from the smartwatch are reported in the MyDignio app.

The investigator must permit study-related monitoring and regulatory reviews, audits, and inspections. The investigators will monitor the data collection to ensure completeness. The study personnel will be closely monitoring the data collection needed for the primary outcome, ASDAS (PROs and CRP), at 6, 12, and 18 months. If data are not reported, the patients will be reminded at least 3 times, either through the MyDignio app, an SMS text message, or a phone call.

### Study Termination

The end of the study was the completion of the 18-month visit. However, patients may withdraw from the study at any time point. Patients with early termination are asked to participate in a study-end visit—a face-to-face visit or a telephone or video consultation. They are then offered regular follow-up at the outpatient clinic as before inclusion in the ReMonit study.

## Results

The project is funded by the Norwegian South-Eastern Regional Health Authority and the Centre for Treatment of Rheumatic and Musculoskeletal Diseases (REMEDY), Diakonhjemmet Hospital, Norway.

Patient screening and recruitment began in September 2021, and inclusion was completed in June 2022. The last 18-month study-end visit will be conducted in December 2023. At present, all included patients were followed up according to the outlined schedule.

## Discussion

### Principal Findings

Patients with IJDs are often followed up in specialist health care for many years. Alternative follow-up strategies with either digital remote monitoring or self-monitoring may be a step toward a more personalized follow-up. By eliminating the need for patients to travel to visits, the ReMonit study aims to enhance accessibility to health care and save valuable time for participants. Furthermore, alternative follow-up strategies may reduce the demands on the health care services [46]. There are few studies evaluating digital follow-up in IJDs [18], and to our knowledge, this trial is among the first to evaluate the effectiveness, satisfaction, safety, and cost-effectiveness of remote digital monitoring and self-monitoring of patient with axSpA.

### Patients

Among the IJDs, we considered patients with axSpA to be the most suitable for remote follow-up and self-monitoring, as disease activity is measured by ASDAS, which is based on PROs together with CRP and not based on a clinical examination. Moreover, treatment is focused on alleviating symptoms. We decided to include only patients treated with TNFi because these patients require follow-up in specialist health care. Furthermore, many patients with axSpA are relatively young, usually without comorbidities, and often experience few side effects of TNFi, which minimizes safety issues. Most patients with axSpA are young and participate in the workforce, making remote follow-up especially attractive in this patient group.

### Single-Center Study

The ReMonit study is a single-center study conducted at the Department of Rheumatology, Diakonhjemmet Hospital, Oslo, Norway. This department is responsible for the largest number of patients with IJDs in Norway. The greater Oslo area is the most densely populated area in Norway. Most patients have short to moderate travel distances to the outpatient clinic. Hence, being allocated to either of the study arms, with face-to-face visits (controls) or remote follow-up (intervention), would be acceptable for most of the screened patients and minimize selection bias at inclusion. The potential benefits of saved costs related to fewer face-to-face visits (transport costs, time use, and absenteeism from work) will probably be greater elsewhere in Norway than in this study.

### Randomization and Blinding

Treating clinicians are not blinded to group allocation. It could have been possible, by having 2 separate HP teams, but would be challenging in terms of study logistics as well as very resource demanding and regarded unfeasible. Blinding of the patients is not possible because the patients need to know the allocated follow-up strategy. However, the patients will not be informed of the primary outcome measures to minimize their influence on the primary outcome. Furthermore, treating clinicians will not know the ASDAS (primary outcome) of the patients during follow-up.

### Study Design

The noninferiority design was chosen because the new follow-up strategies are likely to be less resource demanding (both for patients and HPs) but not be superior to conventional follow-up in maintaining low disease activity. The included patients are well treated with TNFi with an ASDAS <2.1 at inclusion. We cannot expect improvement of disease activity in either of the study arms, and it is unlikely to obtain superiority of the point prevalence of ASDAS <2.1 in the intervention groups. This assumption is based on data from the NOR-DMARD database [42], which showed that 88% of patients with axSpA using TNFi with ASDAS <2.1, remained at ASDAS <2.1 2 years later without change of medication in traditional face-to-face follow-up.

### Primary Outcome

Our main goal is to assess whether these new follow-up strategies are associated with low disease activity over time; hence, it is meaningful to assess the primary outcome, ASDAS, at several time points. There are 2 validated measurements of disease activity of patients with axSpA: ASDAS and BASDAI [27,38]. The ASDAS is the newest and is based on both PROs and objective measurements of inflammation (CRP or ESR) and is the recommended instrument for assessing disease activity when monitoring patients [5,47,48]. Thus, ASDAS was chosen as the primary outcome measure and BASDAI as a secondary outcome measure. Other measures of disease activity are included as secondary objectives together with the measure of functional status measured by the Bath Ankylosing Spondylitis Functional Index, which will provide more comprehensive information about disease activity.

### Secondary Outcomes

We believe that the evaluation of secondary end points in the ReMonit study is important with regard to the clinical implications of the study results. Even if this study shows noninferiority of the primary outcome with the 2 new follow-up strategies, the success of the follow-up strategies is also dependent on patient satisfaction and safety issues, such as adverse events or the use of painkillers. Follow-up with remote monitoring or self-monitoring may not be relevant to implement if there are increased negative effects compared with usual care.

### Selection of Participants

On the basis of prior research and also from clinical experience, where patients at the outpatient clinic often ask for an alternative follow-up rather than face-to-face visits, we hypothesize that patient satisfaction will be high in the remote monitoring and self-monitoring groups [1]. At recruitment, we motivated all patients fulfilling the screening criteria to participate (also patients not interested in digital follow-up) to obtain a representative study sample and avoid selection bias by only recruiting patients especially interested in digital follow-up. It was underscored that patients in all the study arms can ask for a face-to-face visit with a rheumatologist when they need it.

### Safety and Adverse Events

Some patients may experience increased pain or disease activity and a reduced effect of TNFi, and in this case, the patients are instructed to ask for an extra visit. It is possible that some patients in the intervention arms will not ask for an extra visit even when experiencing worsening of the disease and instead increase the use of painkillers. We will register the use of NSAIDs and other painkillers to detect increased use, and we will consider this as a possible adverse event of the intervention. A consequence of increased pain may be reduced sleep quality that will be captured from the reported PROs. The linkage to the Norwegian Prescription Database will provide information on the use of sleeping pills.

### Substudies

Another consequence of not detecting increased disease activity might be increased sick leave. Linkage to FD-trygd (register of work status, sick leave, and disability pension) and patient reports will provide information about possible differences in sick leave between the study arms. As all patients use TNFi, an immunosuppressant, they are at increased risk of infection. We will collect information on the use of antibiotics through patient reports and receive information from the Norwegian Prescription Database to assess whether there are differences in the use of antibiotics between the intervention and control groups.

We hypothesize that patients with remote follow-up and self-monitoring will spend less time on disease-related follow-up. However, there is a possibility that patients need closer follow-up in primary health care when follow-up in specialist health care is less close. Furthermore, fewer prescheduled visits may result in more unscheduled telephone or face-to-face visits. Thus, it is important to analyze the cost-effectiveness of the different follow-up regimen when designing future sustainable follow-up strategies.

Data collection will also provide the opportunity to perform other analyses. Before the implementation of digital follow-up, it is important to explore eHealth literacy and the attitude toward digital follow-up [46]. This will provide important information on facilitators and barriers to remote care and may explain why digital health service implementations work or fail. The qualitative substudy with individual interviews will be conducted after the RCT to gain in-depth insights from patients and HPs on their experiences with remote monitoring. Interviews will explore potential implications for patients’ empowerment, acceptability of remote monitoring, and patients’ views on the frequency and content of remote monitoring. Observations will provide insight into the interaction and communication between patients and HPs with remote follow-up and face-to-face visits.

In arm B, remote monitoring, the patients will self-report on disease activity once a month, and a subgroup will also measure CRP monthly. These data will provide valuable information about how disease activity and inflammation vary over time. The frequent reporting of PROs will provide an opportunity to investigate the optimal frequency of collecting PROs, which is important when designing future follow-up strategies. Frequent registration of disease activity and the tracking of physical activity and heart rate allow for evaluating fluctuations in disease activity and physical activity over time and if flares can be predicted based on changes in monitored physical activity level.

### Conclusions

In conclusion, the ReMonit study will evaluate whether remote digital monitoring or self-monitoring is a clinically effective, safe, and sustainable future follow-up strategy for patients with axSpA. The ReMonit study will contribute to the current knowledge on new follow-up strategies, which may result in improved and more personalized follow-up strategies for patients with axSpA, and potentially lead to reduced costs for the patients and the society.

## Appendix

Multimedia Appendix 1 Aim of the Remote Monitoring in Axial Spondyloarthritis (ReMonit) study.

## Abbreviations

ASDAS: Ankylosing Spondylitis Disease Activity Score
AxSpA: axial spondyloarthritis
BASDAI: Bath Ankylosing Spondylitis Disease Activity Index
CRP: c-reactive protein
DMARD: disease-modifying antirheumatic drug
eCRF: electronic case report form
ESR: erythrocyte sedimentation rate
HP: health care professional
IJD: inflammatory joint disease
NSAID: nonsteroidal anti-inflammatory drug
PGA: Patient Global Assessment
PRO: patient-reported outcome
PsA: psoriatic arthritis
RA: rheumatoid arthritis
RCT: randomized controlled trial
ReMonit: Remote Monitoring in Axial Spondyloarthritis
TNFi: tumor necrosis factor inhibitor

## Data Availability

The full trial protocol (version 1.1; date: 23.12.22) is available from the corresponding author upon request. At present, data sharing is not applicable.
