# Supplementary material for: Follow-Up of Patients With Axial Spondyloarthritis in Specialist Health Care With Remote Monitoring and Self-Monitoring Compared With Regular Face-to-Face Follow-Up Visits (the ReMonit Study): Protocol for a Randomized, Controlled Open-Label Noninferiority Trial
Source: JMIR Res Protoc. 2023 Dec 27;12:e52872. doi: 10.2196/52872 (PMC10782285; doi:10.2196/52872)
Supplement: Multimedia Appendix 1 [file resprot_v12i1e52872_app1.pdf]

ReMonit

Aim of the ReMonit study

To assess 2 new follow-up strategies in patients with axSpA

- Remote monitoring

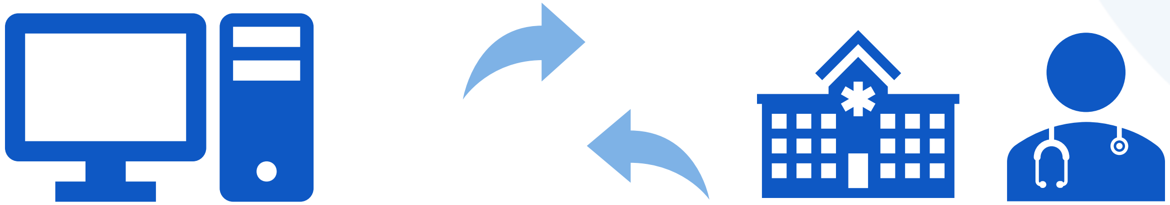

- Self monitoring

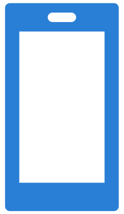

Are either of these strategies associated with
- **Maintaining low disease activity**
- Patient satisfaction
- Safety
- Cost-effectiveness

Compared to conventional prescheduled face-to face visits
